# Genetic diversity, phylogenetic relationships, and marker development between *Hydrangea serrata* and *H. macrophylla* based on plastome and 45S nrDNA

**DOI:** 10.3389/fpls.2026.1836859

**Published:** 2026-08-24

**Authors:** Hyeonah Shim, Jong-Soo Kang, KyoungSu Choi, Saeyeon Hwang, Su Mi Choi, Shin-Jae Kang, Nomar Espinosa Waminal, Hana Lee, Sae Hyun Lee, Jee Young Park, Hyun-Seung Park, Taek Joo Lee, Jung Hwa Kang, Sang Hyun Sung, Ki-Oug Yoo, SeonJoo Park, Tae-Jin Yang

**Affiliations:** 1 Department of Agriculture, Forestry and Bioresources, Plant Genomics and Breeding Institute, Research Institute of Agriculture and Life Sciences, College of Agriculture and Life Sciences, Seoul National University, Seoul, Republic of Korea; 2 Leibniz Institute of Plant Genetics and Crop Plant Research (IPK) Gatersleben, Seeland, Germany; 3 Department of Forest Resources, College of Forest and Environmental Sciences, Kangwon National University, Chuncheon, Republic of Korea; 4 Department of Biology, College of Natural Sciences, Kyungpook National University, Daegu, Republic of Korea; 5 Department of Life Sciences, Yeungnam University, Gyeongsan, Republic of Korea; 6 Department of Applied Plant Sciences, College of Agriculture and Life Sciences, Kangwon National University, Chuncheon, Republic of Korea; 7 Department of Integrative Biological Sciences and Industry, Convergence Research Center for Natural Products, Sejong University, Seoul, Republic of Korea; 8 Hantaek Botanical Garden, Yongin, Republic of Korea; 9 College of Pharmacy and Research Institute of Pharmaceutical Science, Seoul National University, Seoul, Republic of Korea; 10 Department of Biological Sciences, College of Natural Sciences, Kangwon National University, Chuncheon, Republic of Korea; 11 BK21 Agriculture-forestry Bioresource Convergence Center, Seoul National University, Seoul, Republic of Korea; 12 Crop Biotechnology Institute, Institutes of Green-bio Science and Technology, Seoul National University, Pyeongchang, Republic of Korea

**Keywords:** hydrangea, hydrangeaceae, molecular marker, nuclear ribosomal DNA (nrDNA), plastid genome (plastome)

## Abstract

Ornamental hydrangeas (genus *Hydrangea*) are cultivated worldwide for their diverse flower colors and attractive morphology. Here, we assembled the complete plastid genome (plastome) and 45S nuclear ribosomal DNA (45S nrDNA) sequences of 22 individuals representing *H. serrata*, *H. macrophylla*, and related species (*H. arborescens*, *H. paniculata*, *H. petiolaris*, and *H. hydrangeoides*). The plastomes contained up to 2,344 single-nucleotide polymorphisms (SNPs) and 367 insertions/deletions (InDels) within the genus, whereas the assembled 45S nrDNA sequences showed 119 SNPs and 10 InDels. Phylogenetic analyses based on plastome and 45S nrDNA sequences clearly separated *H. serrata* and *H. macrophylla* from the other *Hydrangea* species. In the plastome-based tree, *H. petiolaris* was placed in the same clade as *H. arborescens*, whereas in the 45S nrDNA-based tree it showed a close relationship to *H. hydrangeoides*. The *H. serrata* and *H. macrophylla* samples were not always separated according to their species boundaries, as observed in samples Hse8–Hse12. Notably, one *H. serrata* sample (Hse8), collected from a wild mountainous region of Japan, exhibited a closer genetic relationship to *H. macrophylla* samples, indicating that cultivated hydrangeas may have originated from a specific wild lineage of *H. serrata* adapted to mountainous habitats. Using plastome-derived molecular markers, 66 *Hydrangea* samples were further classified into five groups, with Group II comprising both cultivated *H. macrophylla* and a subset of wild *H. serrata* samples, suggesting a close genetic affinity between this group and the ancestral gene pool of cultivated *H. macrophylla*. Based on these genomic resources, eight plastome-derived molecular markers were developed to differentiate cultivated hydrangeas from wild genotypes and to assess genetic diversity within *H. serrata* and *H. macrophylla*, providing practical tools for germplasm identification, breeding, and genetic resource management of *Hydrangea* species.

## Introduction

The genus *Hydrangea* (family Hydrangeaceae) comprises approximately 70−75 species distributed across southern and eastern Asia, including Korea, China, Japan, the Himalayas, and Indonesia, as well as to the Americas, with the greatest diversity occurring in eastern Asia (McClintock, 1957; Van Gelderen and Van Gelderen, 2004). Most species and cultivars vary in inflorescence color and morphology, contributing to their popularity as ornamental plants (Reed, 2002; Orozco-Obando et al., 2005; Conolly et al., 2010; Samain et al., 2010). Among them, *H. macrophylla*, *H. serrata*, and *H. paniculata* are particularly valued in global horticultural markets (Van Gelderen and Van Gelderen, 2004).

*Hydrangea serrata*, also commonly known as mountain hydrangea or ‘tea of heaven’, is a valuable resource in Korea (Reed and Rinehart, 2007). *Hydrangea serrata* typically occurs in mountainous habitats of Japan and Korea and is characterized by ovate-oblong petals, distinct peduncles, and oblong leaves (Ohba, 2017; Hirota et al., 2022). In contrast, *H. macrophylla* is native mainly to Japan, particularly coastal regions, has obovate to elliptic leaves and a larger nuclear genome (~2,234 Mb) compared to the smaller genome of *H. serrata* (~2,102 Mb) (Kamei et al., 2000; Cerbah et al., 2001; Zonneveld et al., 2005). The close morphological similarity between *H. serrata* and *H. macrophylla* has long complicated their taxonomic delimitation. Example photographs of *H. serrata*, *H. macrophylla* are provided in **Supplementary Figure 1** to illustrate their morphological similarity.

This taxonomic difficulty is reflected in the inconsistent treatment of these taxa in previous studies. *Hydrangea serrata* was previously classified as a subspecies of *H. macrophylla* (McClintock, 1957), and some molecular studies have supported this treatment (Rinehart et al., 2006; Reed and Rinehart, 2007). In contrast, other taxonomic treatments have recognized *H. serrata* and *H. macrophylla* as distinct species based on morphological characters and genome size differences (Van Gelderen and Van Gelderen, 2004; Zonneveld, 2004). Thus, their delimitation remains controversial. In the Korean context, this distinction is particularly relevant because *H. serrata* occurs naturally in Korea, whereas *H. macrophylla* is mainly encountered as cultivated horticultural material. Accurate discrimination between native *H. serrata* germplasm and cultivated *H. macrophylla* or hybrid materials is therefore important for taxonomic identification, conservation of native genetic resources, and future breeding applications.

Plastid genome (plastome) and 45S nuclear ribosomal DNA (nrDNA) sequences provide valuable information for taxonomic identification, genetic diversity analysis, and phylogenetic inference in plants because they contain both conserved and variable regions (Bendich, 1987; Srivastava and Schlessinger, 1991; Álvarez and Wendel, 2003; Wicke et al., 2011; Nguyen et al., 2020; Kang et al., 2023; Lee et al., 2023). Previous studies of *Hydrangea* have used partial plastid genes such as *matK* and *rbcL* and intergenic spacers including *rpl32*–*ndhF*, *trnV*–*ndhC* and *psbT*–*petB* regions, whereas recent complete plastome analyses have provided a broader genus-level framework for phylogenetic relationship within *Hydrangea* (Hufford et al., 2001; Kim et al., 2016; Mendoza et al., 2013; Raman et al., 2023; Yang et al., 2024). Based on this framework, the present study focused primarily on the taxonomically difficult *H. serrata*–*H. macrophylla* clade, while including representative additional *Hydrangea* species as intrageneric comparison taxa for phylogenetic and marker-based analyses. Previous molecular marker studies in *Hydrangea* have used SSR and SNP-based markers for genetic diversity analysis, cultivar identification, population structure analysis, and linkage mapping (Rinehart et al., 2006; Reed and Rinehart, 2007, 2009; Wu and Alexander, 2019, 2020, 2021). However, plastome-derived markers specifically developed for rapid discrimination within the taxonomically difficult *H. serrata*–*H. macrophylla* clade remain limited.

This study aimed to investigate genetic diversity within *Hydrangea* using complete plastome and 45S nrDNA sequences, and to clarify the species boundary between the morphologically similar *H. serrata* and *H. macrophylla*. Samples of *H. serrata* and related species (*H. macrophylla*, *H. paniculata*, *H. arborescens*, *H. petiolaris*, and *H. hydrangeoides*) were collected from diverse locations and analyzed using next-generation sequencing. Comparative analyses of plastomes and 45S nrDNA sequences were conducted to address the taxonomic ambiguity between *H. serrata* and *H. macrophylla*, and eight plastome-derived molecular markers were developed to assess genetic diversity and facilitate species discrimination within *Hydrangea*.

## Materials and methods

### Plant materials

Sixty-six *H. serrata* individuals, ten *H. macrophylla*, three *H. paniculata*, four *H. arborescens*, two *H. petiolaris*, and one *H. hydrangeoides* were obtained from various sources such as wild collections, specimens, and cultivars. A detailed description of all samples used for sequencing and molecular marker validation is listed in **Supplementary Table 1**, including sample origin, sources, and cultivated status. Among the 66 samples, 11 *H. serrata*, five *H. macrophylla*, two *H. arborescens*, two *H. paniculata*, one *H. petiolaris*, and one *H. hydrangeoides* were selected for sequencing and assembly of complete plastome and 45S nrDNA sequences. These samples were selected based on DNA quality and availability, as well as to represent multiple species and groups. The remaining samples, primarily derived from herbarium specimens, were not suitable for high-quality genome sequencing due to DNA degradation.

### DNA extraction and sequencing

Leaf samples were snap-frozen with liquid nitrogen, and then ground with mortars and pestles. Genomic DNA was extracted using GeneAll Plant SV Mini Kit (GeneAll Biotechnology Co. Ltd., Seoul, Korea) following the manufacturer’s protocol. Quantity and quality were examined by gel electrophoresis and NanoDrop ND-1000 (Thermo Scientific Inc., Wilmington, DE, USA). For sequencing, paired-end genomic DNA libraries were constructed by commercial service providers (Lab Genomics, Seongnam, Korea and Phyzen, Seongnam, Korea) and sequenced on Illumina MiSeq and HiSeq platforms (Illumina, USA), using 2 x 300 bp and 2 x 151 bp paired-end reads, respectively (**Supplementary Table 2**). One to 1.5 Gbp of raw data was generated for each sample which were trimmed based on the minimum quality score of 30, using CLC quality trim (ver. 4.06 beta, CLC Inc., Aarhus, Denmark) (**Supplementary Table 2**).

### Assembly and annotation of complete plastomes and 45S nrDNA

A total of 22 *Hydrangea* samples from six species (11 *H. serrata*, five *H. macrophylla*, two *H. paniculata*, two *H. arborescens*, one *H. petiolaris*, and one *H. hydrangeoides*) were assembled using the *de novo* assembly of low-coverage whole-genome sequencing (dnaLCW) method (Kim et al., 2015). In addition, raw sequencing data of *Deutzia scabra* (Hydrangeaceae), used as an outgroup, was downloaded from NCBI GenBank (SRR14570892) and assembled using the same approach. A total of 672 to 1,106 Mbp of trimmed reads were assembled using the CLC genome assembler (ver. 4.06 beta, CLC Inc., Aarhus, Denmark), with the overlap distance set to 150–500 bp. Initial contigs of assembled reads were extracted from total contigs using MUMmer 4 (Marçais et al., 2018) with the *H. serrata* plastome sequence (KU140669), as the reference sequence. Reference sequence matching contigs were arranged and merged by overlapping the sequences. The same method was used for 45S nrDNA assembly with the *H. serrata* nrDNA (OM158458) as the reference sequence. To minimize potential reference bias, plastome assemblies were validated by examining read mapping consistency, including coverage depth, read orientation, and support across junction regions. Annotation was performed using GeSeq v2.03 (Tillich et al., 2017), and the result was manually curated with ARTEMIS v18.2.0 (Carver et al., 2012). Circular maps of the genomes were drawn using OGDRAW v1.3.1 (Greiner et al., 2019).

### Intra- and interspecific diversity and phylogenetic analyses of plastomes and 45S nrDNA sequences

A total of 25 plastome sequences were used for comparative analysis, including 22 newly assembled sequences in this study, one *H. serrata* sequence (Hse7, OL944391) and one *H. macrophylla* sequence (Hma5, OL944390) from a previous study (Raman et al., 2023), and *Deutzia scabra* as the outgroup. For 45S nrDNA, sequences from 24 *Hydrangea* samples and one outgroup species were analyzed. The difference in sample number between the plastome and 45S nrDNA datasets was due to one *H. paniculata* sample for which only the plastome sequence was available in NCBI, with no corresponding 45S nrDNA sequence available.

Multiple sequence alignments of both plastome and 45S nrDNA sequences were performed using MAFFT v7.490 (Katoh and Standley, 2013). Single nucleotide polymorphisms (SNPs) and insertions/deletions (InDels) were identified and counted for each pairwise comparison to assess intra- and interspecific variation. Pairwise nucleotide diversity was calculated from aligned plastome and 45S nrDNA sequences as the number of nucleotide differences divided by the number of comparable aligned nucleotide sites for each sample pair. Sites containing gaps or ambiguous bases in either sequence were excluded, and the outgroup was excluded from nucleotide diversity estimates. The resulting pairwise nucleotide diversity matrices were visualized as heatmaps (**Supplementary Figure 2**).

The best-fit nucleotide substitution model was selected using jModelTest2 based on the AIC criterion, and the GTR+G model was selected for both datasets (Darriba et al., 2012; Guindon and Gascuel, 2003). Phylogenetic analyses were conducted based on the aligned plastome and 45S nrDNA sequences using RAxML v.8.2.12 (Stamatakis, 2014) under the GTRGAMMA substitution model with 1,000 bootstrap replicates.

To examine possible intra-individual heterogeneity of 45S nrDNA copies, raw sequencing reads were mapped back to the assembled 45S nrDNA sequences, and polymorphic sites with mixed nucleotide signals were inspected. For each polymorphic position, the proportion of reads supporting alternative nucleotides was calculated and summarized in **Supplementary Table 4**.

### Development of molecular markers and their application

Both chloroplast and 45S nrDNA sequences were initially evaluated for marker development. However, due to the relatively low level of polymorphism observed in nrDNA regions, variant regions from plastome sequences were selected as candidates for marker development. Markers were developed in three different types: 1) Codominant markers from InDel regions 2) derived cleaved amplified polymorphic sequence (dCAPS) markers, and 3) High-resolution melting (HRM) markers. Markers were designed using Primer-Blast (Ye et al., 2012), Primer3 (Untergasser et al., 2012), and dCAPS Finder 2.0 (Neff et al., 2002). PCR was performed at 25 ul reaction volume containing 1 unit of Taq DNA polymerase (Inclone Biotech, South Korea), 2.5 uL of 10x reaction buffer, 0.2 mM dNTPs, 20 ng genomic DNA, and 10 µM for each forward and reverse primers. PCR conditions were as follows: 94°C for 5 minutes; 35 cycles of denaturation at 94°C for 30 seconds, annealing at 58°C for 30 seconds, elongation at 72°C for 30 seconds; and a final extension at 72°C for 7 minutes. PCR products were confirmed by electrophoresis using 3% (w/v) agarose gel. dCAPS markers were applied with the same PCR conditions mentioned above, using the *AluI* restriction enzyme.

HRM analysis was conducted on a LightCycler 96 Real-time PCR machine (Roche Applied Science, Indianapolis, IN, U.S.A.) in reaction volumes of 20 ul that contains 1 unit of Taq DNA polymerase (Inclone Biotech, South Korea), 2 ul of 10x reaction buffer, 0.2 mM dNTPs, 20 ng genomic DNA, 10 µM for each forward and reverse primers, and 2.5 uM SYTO 9 (Thermo Fisher Scientific, U.S.A.). The PCR conditions were as follows: pre-amplification at 95 °C for 5 minutes; 45 cycles of 95 °C for 20 seconds, 60 °C for 20 seconds, and 72 °C for 20 seconds. After amplification, melting curve analysis was performed by denaturing the PCR products at 95 °C for 1 minute, cooling to 40 °C for 1 minute, then gradually increasing the temperature from 60 °C to 95 °C for HRM analysis with fluorescence acquisition. The reaction was then cooled to 40 °C for 30 seconds. The resulting genotype information were combined to perform cluster analysis with PowerMarker V3.0 (Liu and Muse, 2005).

To further evaluate the marker genotype pattern of *H. macrophylla*, additional publicly available raw-read datasets were obtained from the NCBI SRA database. Seven *H. macrophylla* samples were downloaded as raw sequencing reads and assembled independently using GetOrganelle v1.7.7.0 (Jin et al., 2020). The marker genotypes for the eight plastome-derived regions were assigned in silico based on the nucleotide states at the corresponding marker positions. These public datasets were used for sequence-based validation only and were not included in the experimental PCR/HRM validation because corresponding biological materials were not available.

## Results

### Assembly and annotation of complete plastomes and 45S nrDNA

**Figure 1:**
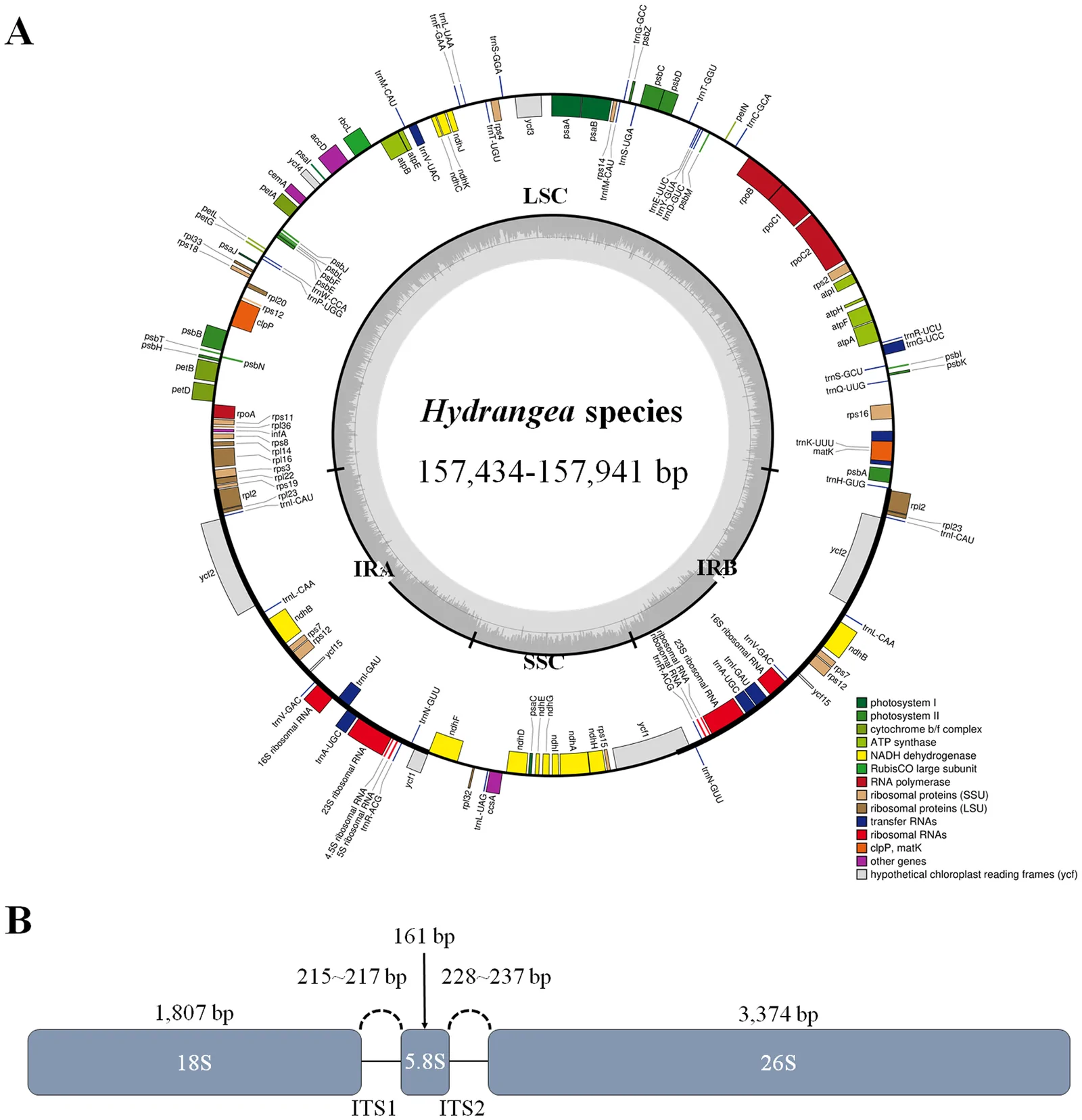
Plastid genome map and 45S nrDNA structure of 22 *Hydrangea* samples newly assembled in this study. **(A)** Circular map of *Hydrangea* plastomes, Total lengths for the *Hydrangea* plastomes assembled in this study are written in the middle of the circle. The outer circle indicates annotated genes in different colors respective to the function information in the legend placed on the bottom right of the figure. **(B)** Arrangement of tandemly repeated 45S nrDNA units in the nuclear genome and their subunits and length. Each box indicates 18S, 5.8S, 26S units from left to right with length ranges written on top. 18S and 5.8S are connected by ITS1 and 5.8S and 26S are separated by ITS2.

Complete plastome sequences were assembled for 22 samples representing six species of the genus *Hydrangea* using the dnaLCW method. All plastomes were assembled into circular form and exhibited a typical quadripartite structure consisting of a large single-copy (LSC) region, a small single-copy (SSC) region, and two inverted repeat (IR) regions (**Figure 1A**). Plastome sizes ranged from 157,434 to 157,941 bp among the assembled samples (**Supplementary Table 3**). The 45S nrDNA unit comprised the 18S, 5.8S, 26S gene clusters separated by the internal transcribed spacer (ITS) regions ITS1 and ITS2 (**Figure 1B**). Complete 45S nrDNA sequences were successfully assembled for all samples, with lengths ranging from 5,782 to 5,794 bp (**Supplementary Table 3**). A total of 114 genes were annotated in the plastomes of all 22 *Hydrangea* samples, including 80 protein-coding genes, 30 transfer RNAs (tRNA), and four ribosomal RNA (rRNA) genes (**Supplementary Table 3**). Gene order was highly conserved across all plastomes, and no structural rearrangements were detected. **Figure 1** is presented as a reference summary of the newly assembled and annotated plastome and 45S nrDNA sequences used in this study. All newly assembled sequences were used for downstream analyses together with previously reported plastome sequences of *H. serrata* (Hse7), *H. macrophylla* (Hma5), *H. paniculata* (NC_044829), and *Deutzia scabra* (SRR14570902) as an outgroup. All plastome and 45S nrDNA sequences generated in this study have been deposited in GenBank, and their accession numbers are provided in **Supplementary Table 3**.

### Intra- and interspecific diversity in plastomes and 45S nrDNA

**Figure 2:**
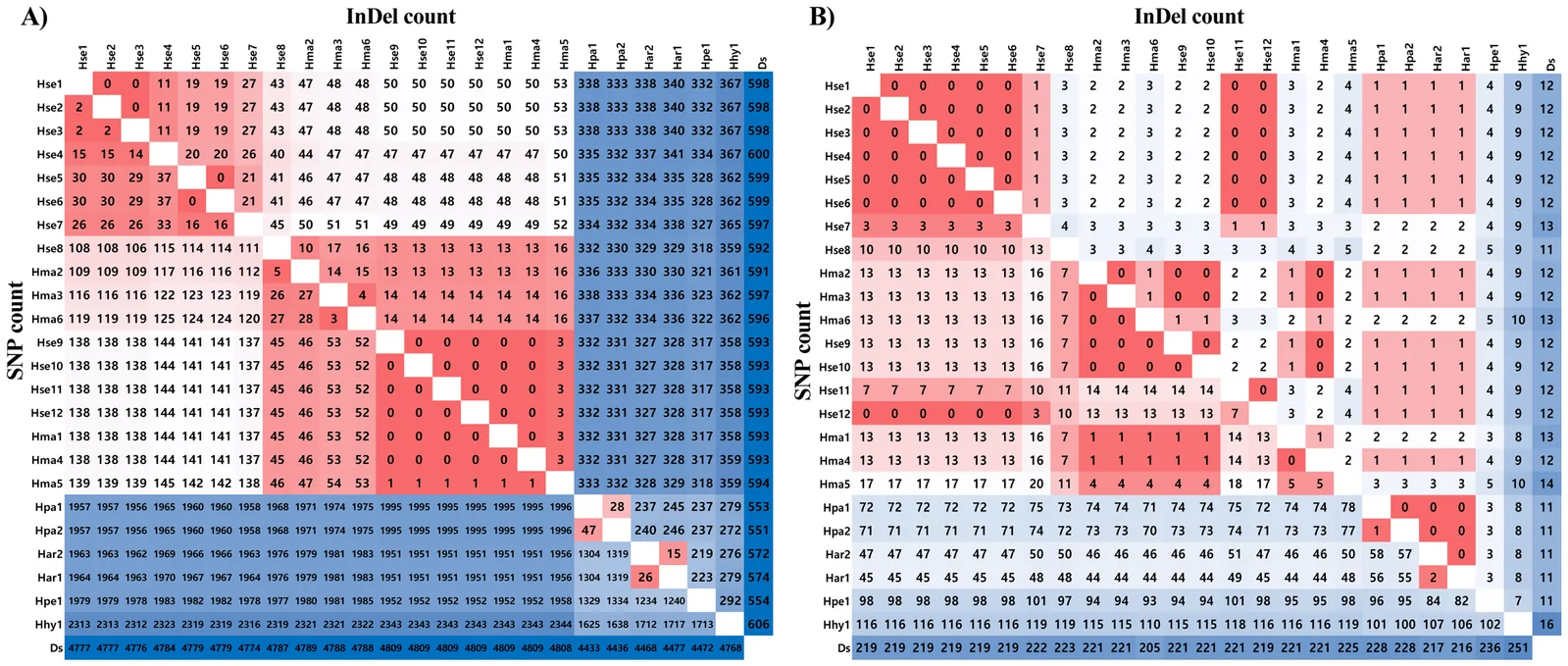
Pairwise comparison matrices of SNP and InDel among 24 *Hydrangea* plastomes and nrDNA sequences. **(A)** Sequence variation detected in *Hydrangea* plastomes. **(B)** Sequence variation detected in *Hydrangea* nrDNA sequences. In each matrix, the lower-left triangle represents the number of SNPs, whereas the upper-right triangle represents the number of InDels. The number of variants for each pairwise comparison is shown in each cell. Color intensity indicates the level of sequence variation, with blue representing higher variation and red representing lower variation.

Pairwise comparisons among *Hydrangea* samples were performed to assess sequence variation based on SNPs and InDels, which were summarized in a matrix (**Figure 2**). Within *H. serrata* and *H. macrophylla* samples, the number of SNPs ranged from 0 to 145 and InDels from 0 to 53, whereas comparisons across the genus revealed up to 2,344 SNPs and 367 InDels. Based on plastome variation, the analyzed samples were divided into three major groups. The first group consisted of *H. serrata* samples (Hse1–7) and showed low levels of variation, with 0–37 SNPs and 0–27 InDels. The second group comprised a mixture of *H. serrata* (Hse8–12) and *H. macrophylla* (Hma1–6) samples and exhibited 0–54 SNPs and 0–17 InDels. The third group included the remaining *Hydrangea* species (*H. paniculata*, *H. arborescens*, *H. petiolaris*, and *H. hydrangeoides*), displaying substantially higher levels of divergence (26–1717 SNPs and 15–292 InDels). Among the samples, Hse5 and Hse6 possessed identical plastome sequences, and Hse10, Hse11, Hse12, Hma1, and Hma4 all shared an identical plastome sequence (**Figure 2**). A corresponding SNP and InDel matrix was constructed for the 45S nrDNA sequences using the same sample order as in the plastome-based analysis. In this analysis, Hse1–7 formed a single group, whereas Hse8–12 which clustered with *H. macrophylla* in the plastome-based comparison, showed greater similarity to the Hse1–7 group. Notably, seven *H. serrata* species (Hse1–6 and Hse12) exhibited identical 45S nrDNA sequences with no detected SNPs or InDels. Across *H. serrata* and *H. macrophylla* samples, overall variation in 45S nrDNA was low, ranging from 0 to 20 SNPs and 0 to 5 InDels. Pairwise nucleotide diversity values calculated from the plastome and 45S nrDNA alignments showed patterns consistent with the SNP/InDel-based comparisons and are provided in **Supplementary Figure 2**.

Artificial hybrid samples may contain heterogeneous 45S nrDNA copies, so read-level nucleotide composition at polymorphic positions in the 45S nrDNA sequences was further inspected. Several samples showed mixed nucleotide signals, indicating the presence of nrDNA variation within the same individual (**Supplementary Table 4**). In particular, Hse9 and the known artificial hybrid Hse10 showed multiple positions with mixed nucleotide states and substantial alternative-read proportions, consistent with the presence of more than one nrDNA variant. Hse11 also showed mixed signals at several positions, whereas Hse12 showed a more homogeneous pattern similar to several non-hybrid *H. serrata* samples. These results indicate that 45S nrDNA sequences in some accessions, especially hybrid individuals, may not be completely homogenized.

### Phylogenetic analyses based on plastome and 45S nrDNA sequences

**Figure 3:**
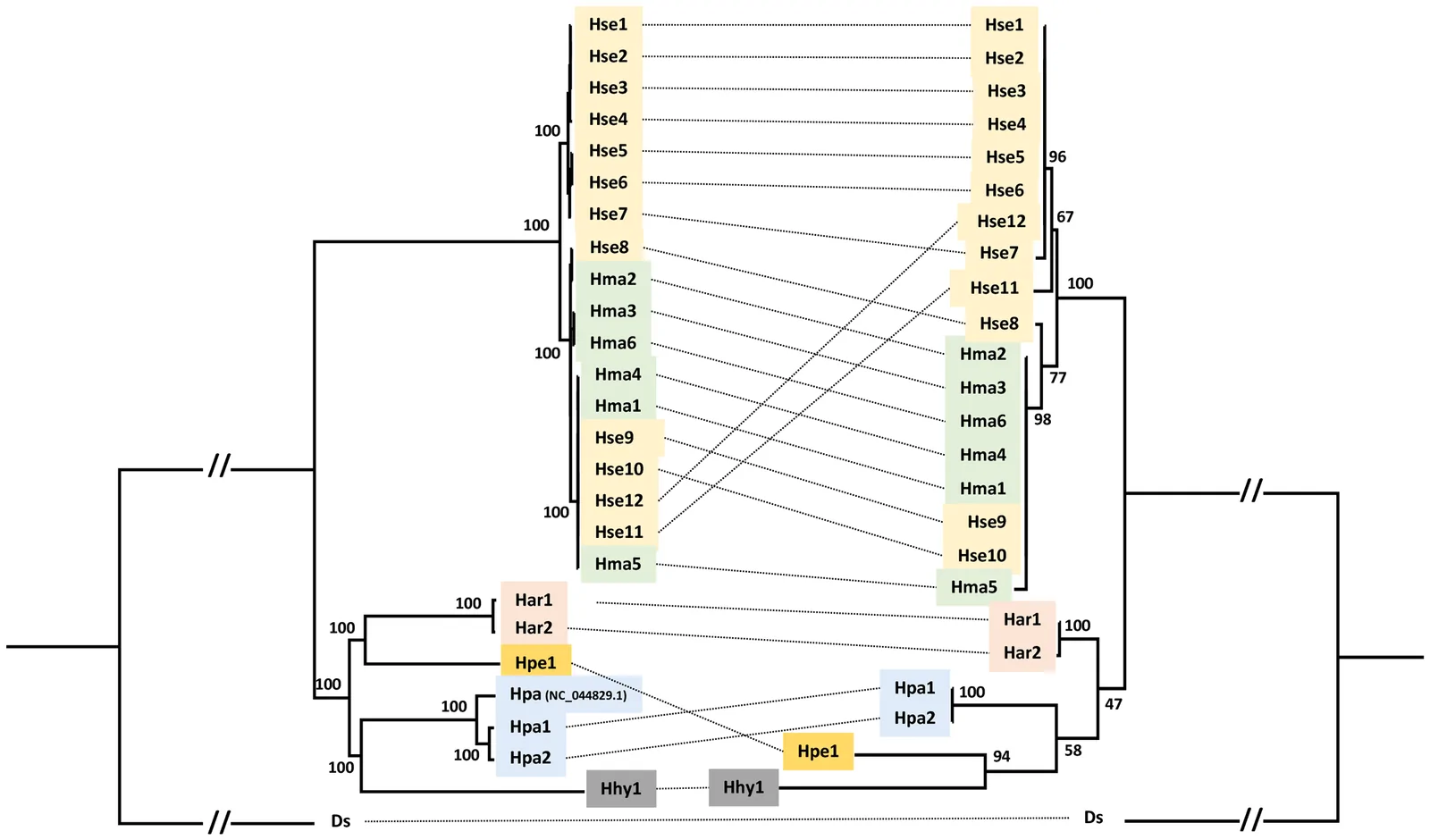
Phylogenetic trees based on plastome (left) and 45S nrDNA sequences (right). *Deutzia scabra* (Ds) was used as the outgroup. Bootstrap values are shown near the branches. Branch lengths are proportional to the estimated number of nucleotide substitutions per site, except for the outgroup branch, which was shortened for visualization. Dotted lines connect the same samples from each phylogenetic tree. Colors represent each species. Yellow: *H. serrata* (Hse), Green: *H. macrophylla* (Hma), Pink: *H. arborescens* (Har), Blue: *H. paniculata* (Hpa), Orange: *H. petiolaris* (Hpe), Gray: *H. hydrangeoides* (Hhy).

Phylogenetic relationships among the *Hydrangea* samples were inferred using plastome and 45S nrDNA sequences, with *Deutzia scabra* as the outgroup (**Figure 3**). The plastome-based phylogeny resolved two major clades. One clade comprised *H. serrata* and *H. macrophylla* samples, whereas the other included *H. arborescens* and *H. petiolaris*, which together formed a sister clade to *H. paniculata* and *H. hydrangeoides*. Within the *H. serrata*–*H. macrophylla* clade, the two species were not clearly separated as five *H. serrata* samples (Hse8, Hse9, Hse10, Hse11, and Hse12) clustered with *H. macrophylla* samples. The 45S nrDNA-based phylogeny also divided the *Hydrangea* samples into two major groups. One group consisted of *H. serrata* and *H. macrophylla* samples, whereas the other comprised *H. paniculata* forming a sister relationship with *H. petiolaris* and *H. hydrangeoides*. Consistent with the plastome-based tree, the nrDNA-based phylogeny did not clearly distinguish *H. serrata* from *H. macrophylla*, with three *H. serrata* samples (Hse8, Hse9, and Hse10) clustering together with *H. macrophylla* samples. Bootstrap support values were generally lower in the 45S nrDNA-based phylogeny compared to the plastome-based tree (**Figure 3**).

To compare the plastome- and nrDNA-based phylogenies, placement patterns of representative accessions across both datasets were summarized (**Table 1**). The comparison revealed both concordant and discordant placements within the *H. serrata*–*H. macrophylla* clade. Hse8–Hse10 clustered with *H. macrophylla* samples in both plastome- and nrDNA-based phylogenies, indicating broadly concordant molecular affinity with *H. macrophylla*. Among these samples, Hse10 is a known artificial hybrid produced in the botanical garden where it were collected. In contrast, Hse11 and Hse12, which are also known artificial hybrids from the same botanical garden, showed discordant placements between the two datasets. In the plastome-based phylogeny, Hse11 and Hse12 clustered with *H. macrophylla* with strong support (BS = 100). In the nrDNA-based phylogeny, however, Hse12 clustered with the other *H. serrata* samples with strong support (BS = 96), and this clade was further grouped with Hse11 with moderate support (BS = 67) (**Figure 3**). These results show that known hybrid samples can display either concordant or discordant plastome/nrDNA placements, highlighting cytonuclear complexity within the *H. serrata*–*H. macrophylla* clade.

**Table 1:** Comparison of plastome- and 45S nrDNA-based phylogenetic placements of *H. serrata* and *H. macrophylla* samples.

Sample	Status	Plastome-based placement	45S nrDNA-based placement	Concordance pattern	Interpretation
Hse1-Hse7	*H. serrata*	*H. serrata* clade	*H. serrata* clade	Concordant	Represents typical *H. serrata* molecular pattern
Hse8	Wild-collected *H. serrata*	*H. macrophylla* associated clade	Close to *H. macrophylla* associated clade	Concordant molecular pattern with *H. macrophylla*	Molecular affinity with *H. macrophylla* despite *H. serrata* morphology
Hse9	Cultivated *H. serrata*	*H. macrophylla* associated clade	*H. macrophylla* associated clade	Concordant molecular pattern with *H. macrophylla*	Molecular affinity with *H. macrophylla* despite *H. serrata* morphology
Hse10	Known artificial hybrid from botanical garden	*H. macrophylla* associated clade	*H. macrophylla* associated clade	Concordant molecular pattern with *H. macrophylla*	Hybrid accession with molecular affinity to *H. macrophylla* in both datasets
Hse11-Hse12	Known artificial hybrid from botanical garden	*H. macrophylla* associated clade	*H. serrata* associated clade	Discordant	Cytonuclear discordance consistent with hybrid origin
Hma1-Hma6	Cultivated *H. macrophylla*	*H. macrophylla* associated clade	*H. macrophylla* associated clade	Concordant	Represents the cultivated *H. macrophylla* lineage

### Development of molecular markers and their application

**Figure 4:**
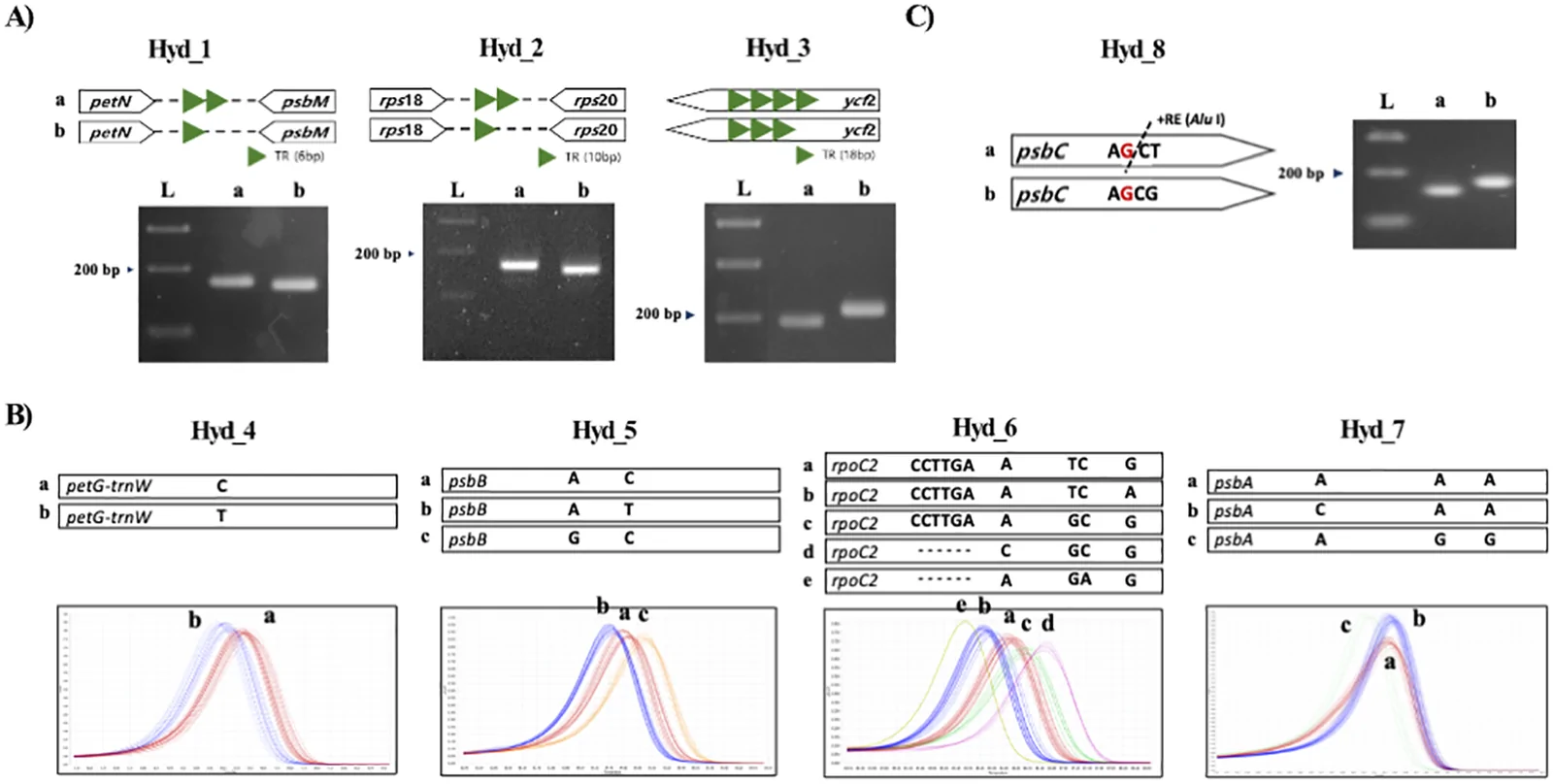
Diagram and genotyping results of molecular markers of *Hydrangea* species developed from the plastome sequence. **(A)** InDel markers developed. **(B)** HRM based markers. **(C)** dCAPS based markers with AluI as the restriction enzyme.

**Figure 5:**
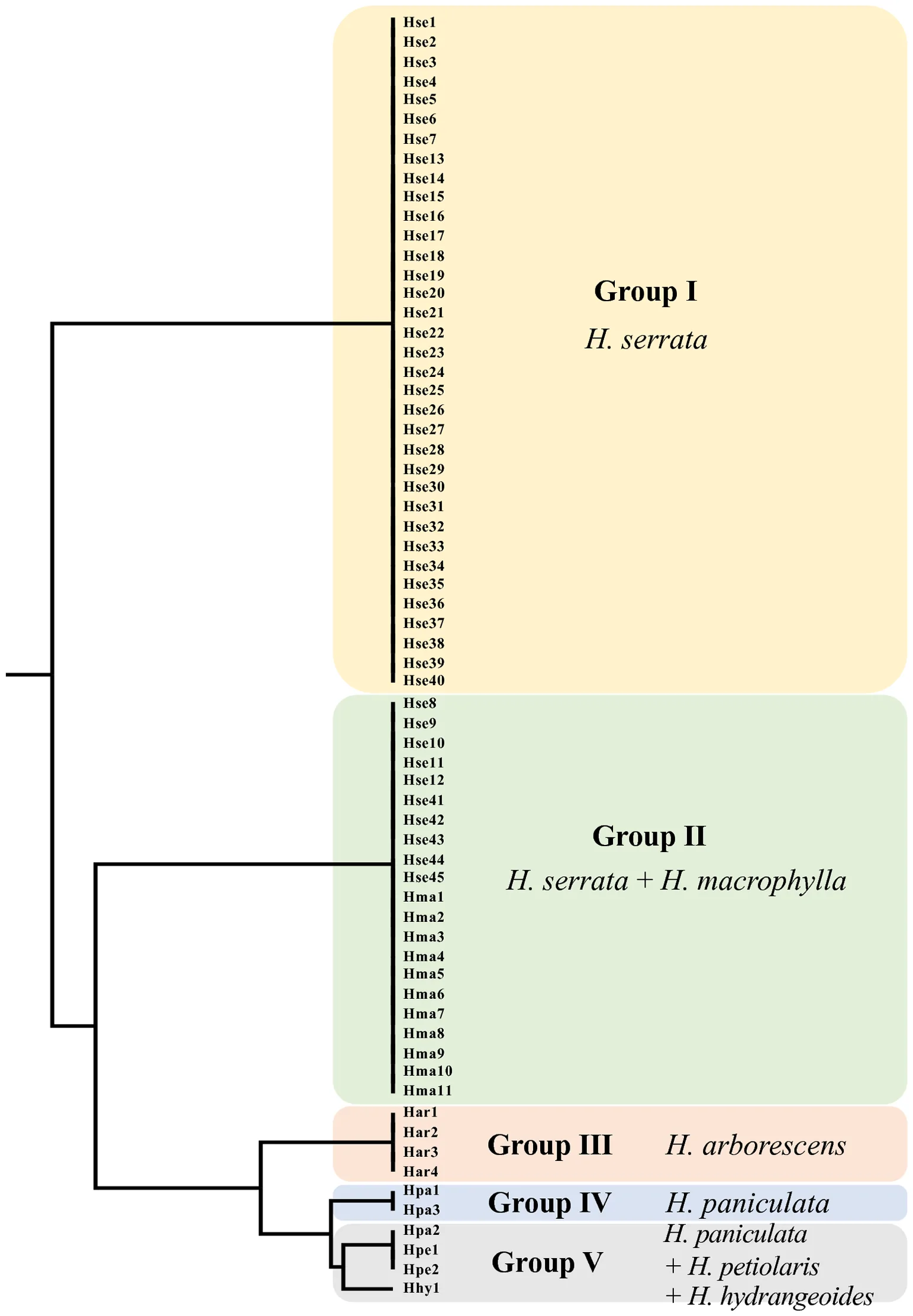
Cluster dendrogram of 66 *Hydrangea* samples based on eight developed molecular markers. The dendrogram was constructed using the UPGMA method based on genetic similarity in PowerMarker. Clusters are indicated by different colors. Yellow: Group 1 – *H. serrata*, Green: Group II – *H. serrata* and *H. macrophylla*, Pink: Group II – *H. arborescens*, Blue: Group IV – *H. paniculata*, Gray: Group V – *H. paniculata*, *H. petiolaris*, *H. hydrangeoides*.

Based on the pairwise alignments of the 24 *Hydrangea* plastome sequences, eight polymorphic regions were selected for marker development to distinguish *Hydrangea* samples. Three codominant markers (Hyd_1, Hyd_2, and Hyd_3) were developed from *petN*-*psbM*, *rps18*-*rpl20*, and *ycf2* regions, respectively (**Table 2**; **Figure 4A**). Four HRM markers (Hyd_4, Hyd_5, Hyd_6, and Hyd_7) were designed from the *petG*-*trnW*, *psbB*, *rpoC2*, and *psbA* regions (**Table 2**; **Figure 4B**), and one dCAPS marker (Hyd_8) was developed from the *psbC* region using the restriction enzyme *Alu*I (**Table 2**; **Figure 4C**). The combination of the eight markers was used to classify the 73 *Hydrangea* samples according to species and genotype. Among the eight markers, Hyd_3 effectively distinguished *H. serrata* and *H. macrophylla* from the other *Hydrangea* species (*H. arborescens*, *H. paniculata*, *H. petiolaris*, and *H. hydrangeoides*). The remaining seven markers further divided the sampled accessions into two groups. The first group comprised Hse1–Hse7 and Hse13–Hse40, whereas the second group comprised Hse8–Hse12, Hse41–Hse45, Hma1–Hma11, and the SRA-derived accessions DRR231908, DRR231924, DRR231925, DRR231927, ERR3361816, ERR12526782, and ERR12526783. Marker-based analyses classified the samples into five genotypic groups (**Figure 5**; **Supplementary Table 5**). Group I consisted of *H. serrata* samples, whereas Group II comprised *H. macrophylla* samples together with ten *H. serrata* samples (Hse8–12 and Hse41–45). Group III included all four *H. arborescens* samples, Group IV contained Hpa1 and Hpa3, and Group V consisted of Hpa2 together with two *H. petiolaris* samples.

**Table 2:** Information of molecular markers developed for the identification of *Hydrangea* species.

Marker type	Marker name	Primer (5’)	Primer (3’)	Annealing temperature (°C)	Amplicon size (bp)	PIC value	Location (plastome)
InDel	Hyd_1	GAAGAAGAACGGACCCCTTT	ATACGTGACTCGCCCAAGAC	58°C	175-181	0.367	*petN*-*psbM*
	Hyd_2	CCAGATTTGATTGTCGTGTTG	AGAATGAACTCCGGGAAGGT	58°C	155-169	0.479	*rps18*-*rpl20*
	Hyd_3	CGGTATTTCTGAACAAGTTCCTGG	TCGGTCTATTTCCGGCATGA	58°C	200-218	0.224	*ycf2*
HRM	Hyd_4	ACGTGGTGATCAGTTGGACC	TGGGTCTCCAAAACCCGATG	58°C	196	0.374	*petG*-*trnW*
	Hyd_5	CGTGCTCAATTGGGTGAAA	AATGCTCCAAATTCCACTTG	58°C	218	0.520	*psbB*
	Hyd_6	GGTTTCCAAAGGAATGTTGT	TAGCAAAAGCCGCTCTCC	58°C	152-158	0.544	*rpoC2*
	Hyd_7	CGGCCAAAATAACCATGA	TCTAGGAATCTCTGGTACTTTCAAC	58°C	252	0.520	*psbA*
dCAPS	Hyd_8	AAGTCATTTTTGGAGGAGAAGC	CCACGGAATTTAAAGAACCTA	58°C	169-180	0.340	*psbC*

The level of polymorphism of the eight developed markers was evaluated using polymorphism information content (PIC) values, calculated from allele frequencies derived from the genotyping results of 66 *Hydrangea* samples (**Supplementary Table 5**). PIC values ranged from 0.224 to 0.544, indicating variable discriminatory power among markers. Among them, Hyd_6 exhibited the highest PIC value (0.544), followed by Hyd_5 and Hyd_7 (0.520), and Hyd_2 (0.479), suggesting these markers are highly informative for distinguishing *Hydrangea* genotypes. In contrast, Hyd_3 showed the lowest PIC value (0.224), reflecting its limited allelic variation despite its effectiveness in separating major species groups. The remaining markers (Hyd_1, Hyd_4, and Hyd_8) displayed moderate levels of polymorphism (PIC = 0.340-0.374). Overall, the combined use of these markers provided sufficient resolution for genotype classification and species differentiation in *Hydrangea*.

## Discussion

*Hydrangea serrata* is primarily distributed in mountainous regions and is considered to be less domesticated, whereas *H. macrophylla* occurs mainly in coastal areas of Japan and has a long history of cultivation and breeding (Van Gelderen and Van Gelderen, 2004; Joung et al., 2010). Previous phylogenetic studies of *Hydrangea* have largely relied on partial plastid regions, such as *matK*, *rbcL*, and *rpl32*-*ndhF*, to infer phylogenetic relationships (Hufford et al., 2001; Mendoza et al., 2013; Kim et al., 2016; Yang et al., 2024). With advances in sequencing technologies, complete plastome sequences have become increasingly accessible, allowing more robust resolution of phylogenetic relationships among closely related species. In this context, a recent study used complete plastome data to investigate evolutionary relationships within *Hydrangea* at the genus level, providing a comprehensive framework for phylogenetic inference in the Hydrangeeae tribe (Raman et al., 2023; Yang et al., 2024).

The taxonomic delimitation between *H. serrata* and *H. macrophylla* has long been debated because of their close morphological similarity despite differences in their typical habitats and distributions: *H. serrata* occurs mainly in mountainous inland habitats, whereas *H. macrophylla* is primarily associated with coastal areas and cultivated horticultural settings in Japan (Rinehart et al., 2006; Reed and Rinehart, 2007; Uemachi et al., 2014). Historically, *H. serrata* was treated as a subspecies of *H. macrophylla* (McClintock, 1957), and this treatment has been supported by several studies based on molecular evidence (Rinehart et al., 2006; Reed and Rinehart, 2007). In contrast, other taxonomic treatments have recognized the two taxa as separate species, primarily on the basis of differences in genome size (Van Gelderen and Van Gelderen, 2004; Zonneveld, 2004). However, genome size variation has been reported within single plant species, suggesting that genome size alone should not be regarded as a sufficient criterion for species delimitation (Goff et al., 2002; Yu et al., 2001; Liu et al., 2014; Parkin et al., 2014).

In this study, several plants morphologically identified as *H. serrata* based on diagnostic characters, including leaf shape and inflorescence morphology, were placed together with *H. macrophylla* at the molecular level, highlighting the incomplete genetic separation between the two taxa. This pattern is biologically meaningful because it suggests that morphological differentiation, ecological distribution, and molecular divergence are not fully concordant in the *H. serrata*–*H. macrophylla* complex. In other words, individuals that can be assigned to *H. serrata* based on morphology and habitat do not always form a distinct molecular lineage from cultivated *H. macrophylla*. This incomplete separation indicates that the evolutionary boundary between the two taxa may be porous rather than sharply defined.

Notably, Hse8 was collected as a wild individual from a mountainous habitat in Japan, preserved as herbarium material, and morphologically identified as typical *H. serrata* (**Supplementary Figure 1**). Despite this identification, both plastome- and nrDNA-based phylogenetic analyses placed Hse8 together with cultivated *H. macrophylla* samples. Therefore, its placement should not be interpreted as evidence of confirmed artificial hybrid origin, but rather as evidence of close genetic affinity that may reflect historical introgression, retained ancestral polymorphism, local geographic structure within wild *H. serrata*, or the contribution of wild *H. serrata*-related lineages to cultivated *H. macrophylla*. This pattern was consistently observed in the molecular marker-based analyses, indicating a close genetic affinity between Hse8 and cultivated *H. macrophylla* samples (**Figure 5**). Marker-based classification of the expanded dataset further showed that Group II comprised a mixture of *H. serrata* and *H. macrophylla* samples, supporting the presence of genetically intermediate or admixed lineages.

Geographic origin and sampling provenance should also be considered when interpreting these marker-based groups. All non-hybrid *H. serrata* samples included in this study have documented origin information, whereas Hse10–Hse12 are known artificial hybrids. In contrast, *H. macrophylla* and other *Hydrangea* samples were cultivars obtained from the Netherlands and maintained in the botanical garden; therefore, their current source does not necessarily represent their original wild provenance. Previous RAPD-based analyses reported geographic structuring within wild *H. serrata* populations (Uemachi et al., 2014). However, because the present study focused on species-boundary assessment using plastome, 45S nrDNA, and plastome-derived markers, these data are not intended to resolve fine-scale geographic structure within wild *H. serrata*. Therefore, Group I and Group II should be interpreted as molecular marker-based groups that may be influenced by geographic origin, but also by hybrid status, cultivation history, and sampling provenance. Recent taxonomic studies have suggested additional complexity within *H. serrata* sensu lato and related taxa in sect. Macrophyllae (Murakami et al., 2024). The Japanese-collected samples analyzed here were morphologically examined and retained as typical *H. serrata* under the taxonomic framework used in this study; thus, their unexpected molecular affinity with *H. macrophylla* further highlights the difficulty of delimiting these closely related taxa.

The mixed composition of Group II may be explained by several non-mutually exclusive evolutionary processes. First, historical hybridization between *H. serrata* and *H. macrophylla* may have promoted introgression, resulting in accessions with genetic affinities to both taxa. Hybridization between *H. serrata* and *H. macrophylla* has been reported previously (Khaing et al., 2018), and the known artificial hybrids included in this study further support the relevance of hybridization in this species complex. Such introgression could blur the molecular boundary between morphologically recognized *H. serrata* and cultivated *H. macrophylla*. Second, incomplete lineage sorting may also have contributed to the observed pattern if the two taxa diverged recently and retained ancestral polymorphisms that have not yet been fully sorted between lineages. Third, the observed pattern may reflect continuing or recurrent gene flow, particularly in regions where wild, semi-wild, and cultivated hydrangeas occur in proximity. Although the present data cannot distinguish conclusively between recent divergence, historical introgression, and ongoing gene flow, the incomplete separation between *H. serrata* and *H. macrophylla* suggests that reproductive isolation between the two taxa may be incomplete.

The known artificial hybrids Hse10–Hse12, although lacking detailed records of exact parental lineages, provide useful examples for interpreting plastome–nrDNA incongruence. Hse10 clustered with *H. macrophylla* in both plastome- and nrDNA-based phylogenies, whereas Hse11 and Hse12 showed discordant placements between the two datasets. This contrast indicates that hybrid origin does not necessarily produce a single phylogenetic pattern; rather, the observed placement may depend on maternal lineage, nuclear inheritance, nrDNA homogenization, and subsequent backcrossing or breeding history. Therefore, the discordance observed in Hse11 and Hse12 is consistent with their known hybrid origin, whereas similar patterns in accessions without documented hybrid status should be interpreted cautiously as possible consequences of incomplete lineage sorting, historical introgression, limited phylogenetic resolution, or sampling effects. The read-level inspection of 45S nrDNA further showed that some hybrid accessions, particularly Hse10, contained mixed nucleotide signals at multiple positions, suggesting that their nrDNA arrays may retain heterogeneous parental or ancestral variants rather than being completely homogenized. Therefore, nrDNA-based phylogenetic placement of hybrid accessions should be interpreted cautiously because the consensus sequence may not fully represent all nrDNA variants present within an individual.

The 45S nrDNA-based phylogeny showed generally lower bootstrap support compared to the plastome-based tree, indicating reduced resolution and lower confidence in some inferred relationships. Importantly, the observed incongruence between plastome- and nrDNA-based phylogenies represents a key finding of this study and suggests a complex evolutionary history underlying these taxa. This discordance is biologically informative because plastome sequences mainly reflect cytoplasmic, usually maternal, lineage history, whereas 45S nrDNA represents a nuclear genomic component. Therefore, disagreement between these two datasets suggests that the cytoplasmic and nuclear histories of some accessions may not be fully concordant. Such cytonuclear discordance is widely documented in plants and is often attributed to multiple evolutionary processes (Rieseberg and Soltis, 1991; Wendel and Doyle, 1998; Rose et al., 2021). First, chloroplast capture through historical hybridization and repeated backcrossing can result in discordant plastid and nuclear phylogenies, and is considered one of the most common causes of topological incongruence in plants (Rieseberg and Soltis, 1991; Okuyama et al., 2005; Huang et al., 2014). In the present study, this process could explain cases in which accessions show affinity to one taxon in the plastome tree but are not resolved in the same way by nrDNA. Second, incomplete lineage sorting (ILS), caused by the persistence of ancestral polymorphisms across speciation events, may also generate discordant gene trees, particularly in recently diverged or rapidly radiating lineages (Maddison, 1997; Degnan and Rosenberg, 2009). This explanation is relevant to the *H. serrata*–*H. macrophylla* complex because their close relationship and incomplete molecular separation suggest that lineage divergence may have been recent or not fully sorted. Third, historical introgression between *H. serrata* and *H. macrophylla*, involving repeated gene flow following hybridization, may have contributed to the complex genetic structure observed in *Hydrangea* (Arnold, 1997). Finally, the extensive artificial breeding history of *Hydrangea*, including recurrent selection and hybridization for ornamental traits, may have further reshaped genomic composition and contributed to cytonuclear discordance and admixture patterns observed in cultivated and semi-wild populations. These processes are not mutually exclusive and may have acted synergistically to produce the observed phylogenetic incongruence. However, because the present study is based on plastome, 45S nrDNA, and selected molecular markers, genome-wide nuclear data will be required to distinguish the relative contributions of chloroplast capture, incomplete lineage sorting, and introgression more rigorously.

These results also have implications for the domestication history of *H. macrophylla*. The close genetic affinity between the wild-collected *H. serrata* sample Hse8 and cultivated *H. macrophylla* samples is consistent with the hypothesis that cultivated hydrangeas may have originated, at least in part, from selected wild lineages related to *H. serrata* with horticulturally favorable traits (McClintock, 1957). Under this scenario, domestication and breeding of *H. macrophylla* may have involved the selection of wild or semi-wild lineages with favorable ornamental traits, followed by artificial selection, clonal propagation, and repeated hybridization. This process could have contributed to the genetic continuum observed between *H. serrata* and *H. macrophylla* in this study.

Complete plastome and 45S nrDNA sequences provide valuable resources for the development of molecular markers useful for species identification and genetic characterization in plants (Park et al., 2019, 2022; Nguyen et al., 2020; Lee et al., 2023; Kim et al., 2025; Joh et al., 2025). Using these genomic resources, we developed eight plastome-derived molecular markers and experimentally validated their utility using 66 *Hydrangea* samples representing the focal *H. serrata*–*H. macrophylla* complex and related species. Although the *H. macrophylla* samples experimentally analyzed in this study were cultivated materials, additional publicly available *H. macrophylla* SRA datasets showed the same genotype pattern across the eight plastome-derived marker regions. This sequence-based comparison further supports the consistency of the *H. macrophylla* marker profile, although broader experimental validation using wild-collected materials would still be valuable when such materials are available. Compared with previously used SSR and SNP-based markers in *Hydrangea*, the plastome-derived markers developed here provide a simple PCR/HRM-based screening tool for classifying the sampled accessions and detecting genetic structure within the *H. serrata*–*H. macrophylla* clade. Within this taxonomically curated panel, the markers classified the samples into five distinct genotypic groups and distinguished *H. serrata* and *H. macrophylla* from other *Hydrangea* species. Given the perennial life cycle of hydrangeas, which requires approximately two years for reliable morphological evaluation (Waki et al., 2018), the application of molecular markers offers a practical alternative for the early identification of breeding materials and parental resources for hybridization (Kardos et al., 2009). The marker system developed in this study provides a useful foundation for future taxonomic, evolutionary, and breeding-oriented studies of *Hydrangea* species. Further validation using additional wild-collected, taxonomically verified materials will help assess their broader applicability across the genus.

## Data Availability

All the assembled sequences are deposited in NCBI under the corresponding accession numbers provided in the **Supplementary Material**.
