# Supplementary material for: Genetic diversity, phylogenetic relationships, and marker development between Hydrangea serrata and H. macrophylla based on plastome and 45S nrDNA
Source: Front Plant Sci. 2026 Aug 24;17:1836859. doi: 10.3389/fpls.2026.1836859 (PMC13547455; doi:10.3389/fpls.2026.1836859)
Supplement: Supplementary file 1 [file DataSheet1.docx]

**Table S1. Information of *Hydrangea* samples collected from various sources.** Asterisk indicates the sample used for sequencing in this study.

| **No.** | **Species Name** | **Sample Name** | **Cultivar Name** | **Sources** | **Origin** | **Status** |
| --- | --- | --- | --- | --- | --- | --- |
| 1 | ***H. serrata*** | Hse1* | Hallasan | Hantaek Botanical Garden, Yongin, South Korea | Netherlands | Cultivated |
| 2 |  | Hse2* | Tamra | Hantaek Botanical Garden, Yongin, South Korea | Jeju Island, South Korea | Cultivated |
| 3 |  | Hse3* | - | Collected | Mt. Hallasan (1200m), Jeju Island, South Korea | Wild |
| 4 |  | Hse4* | - | Collected | Mt. Hallasan (1500m), Jeju Island, South Korea | Wild |
| 5 |  | Hse5* | - | Collected | Mt. Hallasan (610m), Jeju Island, South Korea | Wild |
| 6 |  | Hse6* | - | Collected | Mt. Hallasan (1890m), Jeju Island, South Korea | Wild |
| 7 |  | Hse7 | - | Provided by Yeungnam University | Mt. Palgongsan, Gyeongsangbuk-do, South Korea | Wild |
| 8 |  | Hse8* | - | Hallym University Herbarium | Mt. Nantai, Tsukuba, Japan | Wild |
| 9 |  | Hse9* | - | Hantaek Botanical Garden, Yongin, South Korea | Netherlands | Cultivated |
| 10 |  | Hse10* | - | Hantaek Botanical Garden, Yongin, South Korea | Hybridization within Hantaek Botanical Garden | Cultivated |
| 11 |  | Hse11* | - | Hantaek Botanical Garden, Yongin, South Korea | Hybridization within Hantaek Botanical Garden | Cultivated |
| 12 |  | Hse12* | - | Hantaek Botanical Garden, Yongin, South Korea | Hybridization within Hantaek Botanical Garden | Cultivated |
| 13 |  | Hse13 | - | Collected | Hallasan (860m), Jeju Island, South Korea | Wild |
| 14 |  | Hse14 | - | Collected | Hallasan (910m), Jeju Island, South Korea | Wild |
| 15 |  | Hse15 | - | Collected | Hallasan (1020m), Jeju Island, South Korea | Wild |
| 16 |  | Hse16 | - | Collected | Hallasan (1140m), Jeju Island, South Korea | Wild |
| 17 |  | Hse17 | - | Collected | Hallasan (1220m), Jeju Island, South Korea | Wild |
| 18 |  | Hse18 | - | Collected | Hallasan (1390m), Jeju Island, South Korea | Wild |
| 19 |  | Hse19 | - | Collected | Hallasan (1390m), Jeju Island, South Korea | Wild |
| 20 |  | Hse20 | - | Collected | Hallasan (1390m), Jeju Island, South Korea | Wild |
| 21 |  | Hse21 | - | Collected | Hallasan (1770m), Jeju Island, South Korea | Wild |
| 22 |  | Hse22 | - | Hantaek Botanical Garden, Yongin, South Korea | Jeju Island, South Korea | Cultivated |
| 23 |  | Hse23 | - | Hantaek Botanical Garden, Yongin, South Korea | Jeju Island, South Korea | Cultivated |
| 24 |  | Hse24 | - | Hantaek Botanical Garden, Yongin, South Korea | Jeju Island, South Korea | Cultivated |
| 25 |  | Hse25 | - | Hantaek Botanical Garden, Yongin, South Korea | Jeju Island, South Korea | Cultivated |
| 26 |  | Hse26 | - | Hantaek Botanical Garden, Yongin, South Korea | Jeju Island, South Korea | Cultivated |
| 27 |  | Hse27 | Pink Petticoat | Hantaek Botanical Garden, Yongin, South Korea | Netherlands | Cultivated |
| 28 |  | Hse28 | - | Collected | Neung-Gyeongbong, Gangneung-si, Gangwon-do, South Korea | Wild |
| 29 |  | Hse29 | - | Collected | Mt. Daedunsan, Wanju-gun, Jeollabuk-do, South Korea | Wild |
| 30 |  | Hse30 | - | Collected | Mt. Moaksan, Jeollabuk-do, South Korea | Wild |
| 31 |  | Hse31 | - | Collected | Neung-Gyeongbong, Gangneung-si, Gangwon-do, South Korea | Wild |
| 32 |  | Hse32 | - | Collected | Tokt'ae-san, Jinan-gun, Jeollabuk-do, South Korea | Wild |
| 33 |  | Hse33 | - | Kangwon National Unviersity Herbarium | Mt. Baeksoksan, Pyeongchang-gun, Gangwon-do, South Korea | Wild |
| 34 |  | Hse34 | - | Kangwon National Unviersity Herbarium | Mt. Waryongsan, Andong-si, Gyeongsangbuk-do, South Korea | Wild |
| 35 |  | Hse35 | - | Hallym University Herbarium | Mt. Gongen, Kyushu, Japan | Wild |
| 36 |  | Hse36 | - | Hallym University Herbarium | Mt. Gongen, Kyushu, Japan | Wild |
| 37 |  | Hse37 | - | Hallym University Herbarium | Tokyo, Japan | Wild |
| 38 |  | Hse38 | - | Hallym University Herbarium | Daisen, Japan | Wild |
| 39 |  | Hse39 | - | Hallym University Herbarium | Mt. Yumyeongsan, Gyeonggi-do, South Korea | Wild |
| 40 |  | Hse40 | - | Collected | Jechon-si, Chungcheongbuk-do, South Korea | Wild |
| 41 |  | Hse41 | - | Hantaek Botanical Garden, Yongin, South Korea | Hybridization within Hantaek Botanical Garden | Cultivated |
| 42 |  | Hse42 | - | Hantaek Botanical Garden, Yongin, South Korea | Hybridization within Hantaek Botanical Garden | Cultivated |
| 43 |  | Hse43 | - | Hantaek Botanical Garden, Yongin, South Korea | Hybridization within Hantaek Botanical Garden | Cultivated |
| 44 |  | Hse44 | Grayswood | Hantaek Botanical Garden, Yongin, South Korea | Netherlands | Cultivated |
| 45 |  | Hse45 | - | Hallym University Herbarium | Mt. Gongen, Kyushu, Japan | Wild |
| 46 | ***H. macrophylla*** | Hma1* | - | Hantaek Botanical Garden, Yongin, South Korea | Netherlands | Cultivated |
| 47 |  | Hma2* | Etoile Violette | Hantaek Botanical Garden, Yongin, South Korea | Netherlands | Cultivated |
| 48 |  | Hma3* | Hobella | Hantaek Botanical Garden, Yongin, South Korea | Netherlands | Cultivated |
| 49 |  | Hma4* | Renate Steiniger | Hantaek Botanical Garden, Yongin, South Korea | Netherlands | Cultivated |
| 50 |  | Hma5 | - | Simpol nursery, Busan, South Korea (from Yeungnam University) | Unknown | Cultivated |
| 51 |  | Hma6* | - | Hantaek Botanical Garden, Yongin, South Korea | Unknown | Cultivated |
| 52 |  | Hma7 | Merveille Sanguine | Hantaek Botanical Garden, Yongin, South Korea | Netherlands | Cultivated |
| 53 |  | Hma8 | Zeisig | Hantaek Botanical Garden, Yongin, South Korea | Netherlands | Cultivated |
| 54 |  | Hma9 | Sandra | Hantaek Botanical Garden, Yongin, South Korea | Netherlands | Cultivated |
| 55 |  | Hma10 | All Summer Beauty | Hantaek Botanical Garden, Yongin, South Korea | Netherlands | Cultivated |
| 56 |  | Hma11 | Nikko Blue | Hantaek Botanical Garden, Yongin, South Korea | Netherlands | Cultivated |
| 57 | ***H. arborescens*** | Har1* | Emerald Lace | Hantaek Botanical Garden, Yongin, South Korea | Netherlands | Cultivated |
| 58 |  | Har2* | Annabelle | Hantaek Botanical Garden, Yongin, South Korea | Netherlands | Cultivated |
| 59 |  | Har3 | Pincushion | Hantaek Botanical Garden, Yongin, South Korea | Netherlands | Cultivated |
| 60 |  | Har4 | Pink Annabelle | Hantaek Botanical Garden, Yongin, South Korea | Netherlands | Cultivated |
| 61 | ***H. paniculata*** | Hpa1* | Wim's Red | Hantaek Botanical Garden, Yongin, South Korea | Netherlands | Cultivated |
| 62 |  | Hpa2* | Levana | Hantaek Botanical Garden, Yongin, South Korea | Netherlands | Cultivated |
| 63 |  | Hpa3 | - | Hantaek Botanical Garden, Yongin, South Korea | Seoul, South Korea | Cultivated |
| 64 | ***H. petiolaris*** | Hpe1* | - | Hantaek Botanical Garden, Yongin, South Korea | Ulleung-do, South Korea | Cultivated |
| 65 |  | Hpe2 | - | Collected | Hallasan (860m), Jeju Island, South Korea | Wild |
| 66 | ***H. hydrangeoides*** | Hhy1* | - | Hantaek Botanical Garden, Yongin, South Korea | Ulleung-do, South Korea | Cultivated |

**Note:** Hse10-Hse12, Hse41-Hse43 are known artificial hybrids maintained in the botanical garden. Detailed records of their exact parental accessions or parental lineages were not available; therefore, parental origins could not be assigned with confidence.

**Table S2. Sequencing and trimming information of 22 *Hydrangea* samples.**

| **No.** |  | **ID** | **Sequencing platform** | **Raw reads** | **Raw bases (bp)** | **Trimmed Reads** | | **Trimmed bases (bp)** | |
| --- | --- | --- | --- | --- | --- | --- | --- | --- | --- |
| 1 | ***Hydrangea serrata*** | Hse1 | MiSeq | 4,269,422 | 1,282,590,548 | 3,414,835 | 79.98% | 829,746,503 | 64.69% |
| 2 |  | Hse2 | MiSeq | 3,729,524 | 1,120,551,140 | 2,888,846 | 77.46% | 693,008,271 | 61.85% |
| 3 |  | Hse3 | MiSeq | 4,008,644 | 1,204,231,005 | 3,117,594 | 77.77% | 748,264,864 | 62.14% |
| 4 |  | Hse4 | MiSeq | 3,751,964 | 1,127,150,904 | 2,825,235 | 75.30% | 671,693,162 | 59.59% |
| 5 |  | Hse5 | HiSeq | 4,622,556 | 1,391,389,356 | 3,967,583 | 85.83% | 1,014,526,584 | 72.91% |
| 6 |  | Hse6 | HiSeq | 4,397,698 | 1,323,707,098 | 3,898,654 | 88.65% | 1,005,440,263 | 75.96% |
| 7 |  | Hse8 | HiSeq | 4,294,978 | 1,292,788,378 | 3,753,167 | 87.39% | 963,429,999 | 74.52% |
| 8 |  | Hse9 | MiSeq | 3,439,918 | 992,390,849 | 3,011,857 | 87.56% | 759,721,746 | 76.55% |
| 9 |  | Hse10 | MiSeq | 3,494,694 | 1,049,865,481 | 2,809,228 | 80.39% | 681,308,975 | 64.89% |
| 10 |  | Hse11 | HiSeq | 3,342,504 | 1,006,093,704 | 3,061,622 | 91.60% | 833,566,567 | 82.85% |
| 11 |  | Hse12 | HiSeq | 4,018,982 | 1,209,713,582 | 3,700,264 | 92.07% | 1,006,797,947 | 83.23% |
| 12 | ***Hydrangea macrophylla*** | Hma1 | MiSeq | 5,302,188 | 1,595,958,588 | 4,136,544 | 78.02% | 955,655,370 | 59.88% |
| 13 |  | Hma2 | HiSeq | 4,454,674 | 1,340,856,874 | 4,059,932 | 91.14% | 1,105,942,360 | 82.48% |
| 14 |  | Hma3 | HiSeq | 4,361,832 | 1,312,911,432 | 3,954,897 | 90.67% | 1,074,690,432 | 81.86% |
| 15 |  | Hma4 | HiSeq | 4,228,290 | 1,272,715,290 | 3,841,019 | 90.84% | 1,053,939,791 | 82.81% |
| 16 |  | Hma6 | HiSeq | 3,976,366 | 1,196,886,166 | 3,526,361 | 88.68% | 910,710,596 | 76.09% |
| 17 | ***Hydrangea arborescens*** | Har1 | HiSeq | 3,469,690 | 1,044,376,690 | 3,168,655 | 91.32% | 859,588,789 | 82.31% |
| 18 |  | Har2 | HiSeq | 4,157,286 | 1,251,343,086 | 3,797,943 | 91.36% | 1,033,659,216 | 82.60% |
| 19 | ***Hydrangea paniculata*** | Hpa1 | HiSeq | 3,486,950 | 1,049,571,950 | 3,132,226 | 89.83% | 842,771,951 | 80.30% |
| 20 |  | Hpa2 | HiSeq | 3,732,690 | 1,123,539,690 | 3,311,572 | 88.72% | 892,205,720 | 79.41% |
| 21 | ***Hydrangea petiolaris*** | Hpe1 | MiSeq | 4,771,220 | 1,352,305,748 | 3,633,827 | 76.16% | 845,255,390 | 62.50% |
| 22 | ***Hydrangea hydrangeoides*** | Hhy1 | MiSeq | 5,336,664 | 1,510,168,673 | 4,071,150 | 76.29% | 946,032,938 | 62.64% |

**Table S3. Plastome and 45S nrDNA sequences statistics of 22 *Hydrangea* samples newly assembled in this study.**

|  |  |  |  | **Plastome sequence** | | | | | | | | |  | **45S nrDNA sequence** | |
| --- | --- | --- | --- | --- | --- | --- | --- | --- | --- | --- | --- | --- | --- | --- | --- |
| **Scientific name** | **ID** | **Remark** |  | **Size (bp)** | **LSC (bp)** | **SSC (bp)** | **IR (bp)** | **GC content** | **protein coding** | **tRNA** | **rRNA** | **Accession no.** |  | **Size (bp)** | **Accession no.** |
| *Hydrangea serrata* | Hse1 | cv. Hallasan |  | 157730 | 86789 | 18711 | 26115 | 37.88% | 80(8) | 30(7) | 4(4) | KY412462 |  | 5783 | MG428634 |
|  | Hse2 | cv. Tamra |  | 157730 | 86789 | 18711 | 26115 | 37.88% | 80(8) | 30(7) | 4(4) | KU140669 |  | 5783 | MG428641 |
|  | Hse3 | Halla Mt. 1200m |  | 157730 | 86789 | 18711 | 26115 | 37.88% | 80(8) | 30(7) | 4(4) | KY412465 |  | 5783 | MG428635 |
|  | Hse4 | Halla Mt. 1500m |  | 157691 | 86749 | 18712 | 26115 | 37.89% | 80(8) | 30(7) | 4(4) | KY412468 |  | 5783 | MG428636 |
|  | Hse5 | Halla Mt. 610m |  | 157761 | 86825 | 18706 | 26115 | 37.89% | 80(8) | 30(7) | 4(4) | OR501611 |  | 5783 | OR493793 |
|  | Hse6 | Halla Mt. 1890m |  | 157762 | 86826 | 18706 | 26115 | 37.89% | 80(8) | 30(7) | 4(4) | OR501610 |  | 5783 | OR493794 |
|  | Hse8 | Ja1 |  | 157668 | 86719 | 18701 | 26124 | 37.88% | 80(8) | 30(7) | 4(4) | OR501609 |  | 5783 | OR492609 |
|  | Hse9 | Ja1 |  | 157617 | 86672 | 18697 | 26124 | 37.89% | 80(8) | 30(7) | 4(4) | KY412463 |  | 5784 | MG428637 |
|  | Hse10 | Ja2 |  | 157617 | 86672 | 18697 | 26124 | 37.89% | 80(8) | 30(7) | 4(4) | KY412464 |  | 5784 | MG428639 |
|  | Hse11 | pink (5leaves) |  | 157617 | 86672 | 18697 | 26124 | 37.89% | 80(8) | 30(7) | 4(4) | OR501607 |  | 5783 | OR492610 |
|  | Hse12 | mutant |  | 157617 | 86672 | 18697 | 26124 | 37.89% | 80(8) | 30(7) | 4(4) | OR501606 |  | 5783 | OR493795 |
| *Hydrangea macrophylla* | Hma1 | Hm |  | 157617 | 86672 | 18697 | 26124 | 37.89% | 80(8) | 30(7) | 4(4) | MG383373 |  | 5785 | MG428648 |
|  | Hma2 | cv. Etoile Violette |  | 157675 | 86716 | 18699 | 26130 | 37.89% | 80(8) | 30(7) | 4(4) | OM140833 |  | 5784 | OM132192 |
|  | Hma3 | cv. Hobella |  | 157659 | 86718 | 18699 | 26121 | 37.89% | 80(8) | 30(7) | 4(4) | OM140834 |  | 5784 | OM132193 |
|  | Hma4 | cv. Renate Steiniger |  | 157617 | 86672 | 18697 | 26124 | 37.89% | 80(8) | 30(7) | 4(4) | OM140838 |  | 5784 | OM132197 |
|  | Hma6 | Chimera |  | 157661 | 86760 | 18699 | 26101 | 37.89% | 80(8) | 30(7) | 4(4) | OR501608 |  | 5784 | OR493796 |
| *Hydrangea arborescens* | Har1 | cv. Annabelle |  | 157941 | 86883 | 18790 | 26134 | 37.87% | 80(8) | 30(7) | 4(4) | OM140837 |  | 5782 | OM132196 |
|  | Har2 | cv. Emerald Lace |  | 157932 | 86875 | 18789 | 26134 | 37.86% | 80(8) | 30(7) | 4(4) | OM140835 |  | 5782 | OM132194 |
| *Hydrangea paniculata* | Hpa1 | cv. Wim's Red |  | 157796 | 86811 | 18739 | 26123 | 37.79% | 80(8) | 30(7) | 4(4) | OM140832 |  | 5783 | OM132191 |
|  | Hpa2 | cv. Levana |  | 157823 | 86838 | 18739 | 26123 | 37.78% | 80(8) | 30(7) | 4(4) | OM140836 |  | 5783 | OM132195 |
| *Hydrangea petiolaris* | Hpe1 | Hp |  | 157692 | 86721 | 18753 | 26109 | 37.83% | 80(8) | 30(7) | 4(4) | KY412467 |  | 5785 | OM135514 |
| *Hydrangea hydrangeoides* | Hhy1 | Sh |  | 157434 | 86591 | 18703 | 26070 | 37.82% | 80(8) | 30(7) | 4(4) | KY412466 |  | 5794 | OM135575 |

Note: Values in parentheses represent the number of duplicated genes located in the IR regions.

**Table S4. Read-level nucleotide composition at polymorphic sites in 45S nrDNA sequences of *H. serrata* and *H. macrophylla* samples.** Polymorphic positions are shown according to their coordinates in the 45S nrDNA alignment. Nucleotide states indicate the base detected at each position. Mixed nucleotide states, such as C/T or A/G, indicate sites where more than one nucleotide was detected in the mapped reads. Values under mix nucleotide states indicate the proportion of reads supporting the alternative nucleotide at that position. A dash indicates a deletion or absence of the corresponding nucleotide. Hse7 and Hma5 were not included in the analysis because only assembled 45S nrDNA sequences were provided and raw reads were unavailable.

|  | **694** | **1864** | **1869** | **1887** | **1902** | **1935** | **1985** | **1997** | **2004** | **2214** | **2249** | **2250** | **2283** | **2329** | **2330** | **2331** | **2392** | **2875** | **2957** | **3491** | **3518** | **5494** |
| --- | --- | --- | --- | --- | --- | --- | --- | --- | --- | --- | --- | --- | --- | --- | --- | --- | --- | --- | --- | --- | --- | --- |
| **Hse1** | C | T | C | C | C | T | G | T | G | T | T | G | A | C | T | A | G | G | C | T | G | C |
| **Hse2** | C | T | C | C | C | T | G | T | G | T | T | G | A | C | T | A | G | G | C | T | G | C |
| **Hse3** | C | T | C | C | C | T | G | T | G | T | T | G | A | C | T | A | G | G | C | T | G | C |
| **Hse4** | C | T | C | C | C | T | G | T | G | T | T | G | A | C | T | A | G | G | C | T | G | C |
| **Hse5** | C | T | C | C | C | T | G | T | G | T | T | G | A | C | T | A | G | G | C | T | G | C |
| **Hse6** | C | T | C | C | C | T | G | T | G | T | T | G | A | C | T | A | G | G | C | T | G | C |
| **Hse8** | C | C | T | T | C | C | - | T | A | C | T | G | A | C/G 31.5% | T/A 31.1% | A/C 31.5% | A | G | C | C | G | C |
| **Hse9** | C/T 23.5% | C/T 23.5% | C/T 25.7% | C/T 24.4% | A/C 25.5% | C/T 25.0% | -/G 25.3% | C/T 27.1% | A/G 26.1% | C/T 25.4% | T/- 24.2% | G/- 23.4% | A | C | T | A | A/G 22.0% | A/G 21.9% | T/C 19.4% | C/T 20.5% | G | C |
| **Hse10** | C/T 19.3% | C/T 33.7% | C/T 35.5% | C/T 36.0% | A/C 35.0% | C/T 32.5% | -/G 30.4% | C/T 33.0% | A/G 32.7% | C/T 26.9% | T/- 23.8% | G/- 23.1% | A | C | T | A | A/G 28.1% | A/G 37.2% | T/C 34.4% | C/T 35.4% | G | C |
| **Hse11** | C | T | C | C/T 44.6% | C | C/T 47.8% | G | T | G/A 49.0% | T/C 46.1% | T | G | A/G 42.3% | C | T | A | G/A 45.3% | G | C | T/C 43.1% | G | C/T 24.39% |
| **Hse12** | C | T | C | C | C | T | G | T | G | T | T | G | A | C | T | A | G | G | C | T | G | C |
| **Hma1** | C | C | T | T | A | C | - | C/T 42.6% | A | C | T | G | A | C | T | A | A | A | T | C | G | C |
| **Hma2** | C/T 10.6% | C | T | T | A | C | - | C | A | C/T 14.3% | T | G | A | C | T | A | A | A | T | C | G | C |
| **Hma3** | C | C | T | T | A | C | - | C | A | C | T | G | A | C | T | A | A | A | T | C | G/A 24.4% | C |
| **Hma4** | C/T 18.8% | C | T | T | A | C | - | C/T 44.2% | A | C | T | G | A | C | T | A | A | A | T | C | G | C |
| **Hma6** | C/T 11.5% | C | T | T | A | C | - | C | A | C | T | G | A | C | T | A | A | A | T | C | G | C |

**Table S5. Genotyping result using eight molecular markers on 66 *Hydrangea* samples.** Allele codes (A–E) represent distinct allele classes defined based on marker-specific genotyping patterns (e.g., fragment size differences for InDel and dCAPS markers and melting profile clusters for HRM markers). Allele code A is highlighted in red, B in blue, C in green, D in yellow, and E in grey. Sample names beginning with DRR or ERR indicate public raw-read datasets obtained from the NCBI SRA database. For these samples, genotypes were inferred in silico from assembled sequence data rather than determined experimentally by PCR/HRM analysis.

|  | **Codominant (InDel)** | | | **HRM** | | | | | **dCAPS** | |
| --- | --- | --- | --- | --- | --- | --- | --- | --- | --- | --- |
|  | **Hyd_1** | **Hyd_2** | **Hyd_3** | | **Hyd_4** | **Hyd_5** | **Hyd_6** | **Hyd_7** | | **Hyd_8** |
| **Hse1** | A | A | A | | A | A | A | A | | A |
| **Hse2** | A | A | A | | A | A | A | A | | A |
| **Hse3** | A | A | A | | A | A | A | A | | A |
| **Hse4** | A | A | A | | A | A | A | A | | A |
| **Hse5** | A | A | A | | A | A | A | A | | A |
| **Hse6** | A | A | A | | A | A | A | A | | A |
| **Hse7** | A | A | A | | A | A | A | A | | A |
| **Hse8** | B | B | A | | B | B | B | B | | B |
| **Hse9** | B | B | A | | B | B | B | B | | B |
| **Hse10** | B | B | A | | B | B | B | B | | B |
| **Hse11** | B | B | A | | B | B | B | B | | B |
| **Hse12** | B | B | A | | B | B | B | B | | B |
| **Hse13** | A | A | A | | A | A | A | A | | A |
| **Hse14** | A | A | A | | A | A | A | A | | A |
| **Hse15** | A | A | A | | A | A | A | A | | A |
| **Hse16** | A | A | A | | A | A | A | A | | A |
| **Hse17** | A | A | A | | A | A | A | A | | A |
| **Hse18** | A | A | A | | A | A | A | A | | A |
| **Hse19** | A | A | A | | A | A | A | A | | A |
| **Hse20** | A | A | A | | A | A | A | A | | A |
| **Hse21** | A | A | A | | A | A | A | A | | A |
| **Hse22** | A | A | A | | A | A | A | A | | A |
| **Hse23** | A | A | A | | A | A | A | A | | A |
| **Hse24** | A | A | A | | A | A | A | A | | A |
| **Hse25** | A | A | A | | A | A | A | A | | A |
| **Hse26** | A | A | A | | A | A | A | A | | A |
| **Hse27** | A | A | A | | A | A | A | A | | A |
| **Hse28** | A | A | A | | A | A | A | A | | A |
| **Hse29** | A | A | A | | A | A | A | A | | A |
| **Hse30** | A | A | A | | A | A | A | A | | A |
| **Hse31** | A | A | A | | A | A | A | A | | A |
| **Hse32** | A | A | A | | A | A | A | A | | A |
| **Hse33** | A | A | A | | A | A | A | A | | A |
| **Hse34** | A | A | A | | A | A | A | A | | A |
| **Hse35** | A | A | A | | A | A | A | A | | A |
| **Hse36** | A | A | A | | A | A | A | A | | A |
| **Hse37** | A | A | A | | A | A | A | A | | A |
| **Hse38** | A | A | A | | A | A | A | A | | A |
| **Hse39** | A | A | A | | A | A | A | A | | A |
| **Hse40** | A | A | A | | A | A | A | A | | A |
| **Hse41** | B | B | A | | B | B | B | B | | B |
| **Hse42** | B | B | A | | B | B | B | B | | B |
| **Hse43** | B | B | A | | B | B | B | B | | B |
| **Hse44** | B | B | A | | B | B | B | B | | B |
| **Hse45** | B | B | A | | B | B | B | B | | B |
| **Hma1** | B | B | A | | B | B | B | B | | B |
| **Hma2** | B | B | A | | B | B | B | B | | B |
| **Hma3** | B | B | A | | B | B | B | B | | B |
| **Hma4** | B | B | A | | B | B | B | B | | B |
| **Hma5** | B | B | A | | B | B | B | B | | B |
| **Hma6** | B | B | A | | B | B | B | B | | B |
| **Hma7** | B | B | A | | B | B | B | B | | B |
| **Hma8** | B | B | A | | B | B | B | B | | B |
| **Hma9** | B | B | A | | B | B | B | B | | B |
| **Hma10** | B | B | A | | B | B | B | B | | B |
| **Hma11** | B | B | A | | B | B | B | B | | B |
| **DRR231908** | B | B | A | | B | B | B | B | | B |
| **DRR231924** | B | B | A | | B | B | B | B | | B |
| **DRR231925** | B | B | A | | B | B | B | B | | B |
| **DRR231927** | B | B | A | | B | B | B | B | | B |
| **ERR3361816** | B | B | A | | B | B | B | B | | B |
| **ERR12526782** | B | B | A | | B | B | B | B | | B |
| **ERR12526783** | B | B | A | | B | B | B | B | | B |
| **Har1** | B | B | B | | B | C | D | C | | A |
| **Har2** | B | B | B | | B | C | D | C | | A |
| **Har3** | B | B | B | | B | C | D | C | | A |
| **Har4** | B | B | B | | B | C | D | C | | A |
| **Hpa1** | B | C | B | | B | C | C | C | | A |
| **Hpa2** | A | C | B | | B | C | C | C | | A |
| **Hpa3** | B | C | B | | B | C | C | C | | A |
| **Hpe1** | A | C | B | | B | C | C | C | | A |
| **Hpe2** | A | C | B | | B | C | C | C | | A |
| **Hhy1** | A | C | B | | B | C | E | C | | A |

**Figure S1. Representative morphology of *H. serrata*, *H. macrophylla*, and hybrid individuals.** Representative photographs show morphological features used for identification, including leaf shape, inflorescence morphology, and peduncle development. A) Example of *H. serrata* (Hse1), B) *H. macrophylla* (Hma3), C) hybrid individuals representing known artificial hybrids maintained in the botanical garden (Hse10), and D) *H. serrata* originally collected from Mt. Nantai, Tsukuba, Japan, and obtained from the herbarium (Hse8).

**Figure S2. Pairwise nucleotide diversity among *Hydrangea* samples based on plastome and 45S nrDNA alignments.** Heatmaps show pairwise nucleotide diversity calculated from (A) complete plastome and (B) 45S nrDNA alignments. Values represent the number of nucleotide differences per comparable aligned site. Sites with gaps or ambiguous bases were excluded, and the outgroup *Deutzia scabra* was excluded from the calculation.
